# Efficacy and cost-effectiveness of intraoperative cell salvage for patients with placenta previa during cesarean section: a retrospective comparison analysis

**DOI:** 10.1515/med-2026-1404

**Published:** 2026-06-01

**Authors:** Le Zhang, Yaojun Lu, Hanli Hua, Yannan Li, Jing Jiao, Shaoqiang Huang

**Affiliations:** Department of Anesthesiology, Obstetrics and Gynecology, Hospital of Fudan University, Shanghai Key Lab of Reproduction and Development, Shanghai Key Lab of Female Reproductive Endocrine Related Diseases, Shanghai, China

**Keywords:** placenta previa, intraoperative cell salvage, allogeneic blood transfusion, cesarean section, operative time, hemoglobin

## Abstract

**Objectives:**

This study aimed to evaluate whether intraoperative cell salvage (ICS) and retransfusion reduce allogeneic blood transfusion (ABT) requirement and treatment costs in patients with placenta previa undergoing cesarean section.

**Methods:**

We retrospectively enrolled 102 pregnant women who underwent ICS and 113 women who did not. The primary outcomes included ABT rate, ABT volume, routine blood tests, and coagulation function before and 24 h after surgery. Secondary outcomes included the costs of all blood component transfusions. Linear regression analysis was performed to identify independent risk factors for ABT.

**Results:**

No statistically significant difference in ABT rate was found between the ICS and control groups. Linear regression analysis revealed that estimated blood loss (EBL) and operative time were independent risk factors for ABT. Subgroup analysis showed that the overall ABT rate, median ABT volume and median RBC values were significantly different between the ICS group and the control group for patients with EBL <800 mL; in patients with EBL >800 mL, the median RBC transfusion was significantly lower in the ICS group. The median ABT cost was significantly higher in the ICS group than in the control group in patients with EBL <800 mL and operative time <67 min, but no significant difference was observed in other subgroups. Postoperatively, the median hemoglobin and hematocrit levels were significantly higher in the ICS group than in the control group.

**Conclusions:**

ICS may be recommended for placenta previa patients who undergo cesarean section for more than 1 h.

## Introduction

Placenta previa (PP), characterized by an abnormal placenta overlying the endocervical internal ostium, is a frequently encountered obstetric condition in women of advanced age, as well as those with a history of previous abortion or cesarean deliveries [1], 2]. The overall incidence of PP is estimated at 5.2 per 1,000 pregnancies worldwide, with Asian (including Chinese) populations having the highest prevalence (12.2 per 1,000 pregnancies) [3], 4]. Pregnant women with PP are associated with a higher risk of perioperative massive hemorrhage (approximately 20 %) in instances of repeat cesarean section [5], 6] which is a leading cause of maternal deaths (contributing to a quarter of deaths annually) [7], 8]. Blood transfusions are required to alleviate hemodynamic instability and reduce the risk of death [9].

Allogeneic blood transfusion (ABT) is the most common option for blood supplementation. However, ABT use carries a risk of several potential adverse reactions, such as hemolytic, febrile, urticarial, and anaphylactic reactions, and transfusion-transmitted infections [10]. More importantly, blood for transfusion is limited and expensive [10]. To minimize the need for ABT and reduce the treatment cost, recent studies recommend performing intraoperative autologous blood cell salvage (ICS) and transfusion [11], 12]. There is extensive evidence to demonstrate the use of ICS results in a significantly lower requirement for allogeneic blood products in several surgeries [13], [14], [15]. However, evidence for its use in cesarean section remains rare and conclusions are controversial [16]. In one study, Khan et al. found that the donor blood transfusion rate was similar between the ICS group and control (9.6 vs. 8.9 %) in cesarean section for abnormal placentation [17], while Zeng et al. demonstrated the allogeneic red blood cell (RBC) transfusion rate was significantly lower in the ICS group than that in the control group (19.4 vs. 57.4 %) [18]. Furthermore, the median cost of RBC products in the ICS group was found to be slightly lower than the control group in the study of Zeng et al., although no significant difference was observed [18]. Lim et al. previously suggested that cell salvage use for cases at high risk of hemorrhage was cost-effective (incremental cost-effectiveness ratio, 34,881 dollars per quality-adjusted life-year gained) [19]. Therefore, further trials are required to evaluate the efficacy and cost-effectiveness of ICS for patients at high risk for hemorrhage during cesarean section, such as those with PP.

The goal of the present study was to compare the ABT rate, ABT volume, treatment cost, and changes in routine blood and coagulation function in patients with PP who underwent ICS transfusion or not. These results may provide additional evidence for the application of ICS for blood transfusion in patients with PP undergoing cesarean sections.

## Methods

### Participants

This retrospective study enrolled pregnant women with PP who underwent cesarean section at our hospital. Patients were excluded if they had hematological diseases, untreated hypertension (defined as diastolic blood pressure >95 mm Hg), renal or liver dysfunction, or sickle cell anemia. Implementation of the ICS program at our hospital began in May 2016, and patients in the ICS group were enrolled from the start date to July 2017. In addition, control women were enrolled from January 2014 to December 2015, prior to implementation of the ICS system. This study was approved by the Institutional Review Board of our hospital (No. 2025-231, Date: Dec 8, 2025). The requirement for written informed consent was waived due to the retrospective nature of the study.

### Blood transfusion procedure

The cell saver Xtra (Sorin Group, Milan, Italy) was used for cell salvage and autologous blood recovery with a volume of 125 mL. The blood reservoir was primed with 200 mL of heparinized saline solution (30,000 U in 1,000 mL) using the fastest flow rate to prevent coagulation, and the infusion speed was adjusted to 60 drips/min. The blood accumulated in the operation field was suctioned into the blood reservoir with a negative pressure of 300 mmHg, and filtered with a 40-μm filter. Salvaged RBCs were automatically pumped into another blood reservoir. ICS blood was re-transfused into patients according to the following criteria: 1) the product of collected ICS volume and hematocrit (HCT, %) >60 and parturient hemoglobin (Hb) level >10 g/dL; 2) the product of collected ICS volume and HCT >50 and the parturient Hb was >9–10 g/dL; and 3) the product of collected ICS volume and HCT >20 and the parturient Hb was <9 g/dL. ABT was recommended if Hb levels were (still) lower than <7 g/dL.

### Data collection

General clinical characteristics, including age, gestational age, gravidity, parity, scarred uterus, PP type, gestational hypertension, anemia, twins, gestational diabetes mellitus (GDM), eclampsia, advanced age, oligohydramnios, premature delivery, and *in vitro* fertilization (IVF), were collected. The primary outcomes included estimated blood loss (EBL), ICS transfusion volume, ABT volume, routine blood test results (Hb, HCT, RBCs), and coagulation function (D-dimer and fibrinogen) before and 24 h after surgery. Secondary outcomes included the costs of all blood component transfusions.

### Statistical analysis

Statistical analyses were performed using SPSS software (version 18.0; SPSS Inc., Chicago, IL, USA). For continuous variables, normality was checked first using the Kolmogorov-Smirnov test. Normally distributed data were presented as the mean and standard deviation (SD), and were compared using independent Student’s *t*-tests. Non-normally distributed data were expressed as median (interquartile range, IQR) and were compared with the Mann-Whitney U test. Categorical variables are expressed as frequencies and were compared using the chi-square (or Fisher) test. To determine which variables were significantly related to allogenic transfusion volume or postoperative Hb, HCT, and fibrinogen levels, linear regression analyses were conducted using maternal baseline characteristics and intraoperative EBL, preoperative Hb, HCT, fibrinogen, operative time, and autologous transfusion volume as covariate risk factors. Statistical significance was set at p<0.05.

### Ethical approval and consent to participate

This study was approved by the Institutional Review Board of our hospital (No. 2025-231, Date: Dec 8, 2025). The requirement for written informed consent was waived due to the retrospective nature of the study.

## Results

A total of 215 eligible subjects were enrolled after application of inclusion and exclusion criteria, of whom 102 underwent ICS treatment and 113 served as controls. The maternal characteristics were similar between the two groups (Table 1), indicating their comparability.

**Table 1:** Baseline characteristics of patients in the ICS and control groups.

Characteristics	ICS group (n=102)	Control group (n=113)	p-Value
Advanced age	14	13	0.539
Gestational weeks	35.7 ± 2.2	36.3 ± 2.4	0.084
Gravidity ≥2	59	69	0.677
Parity >2	0	1	1.00
Scarred uterus	38	38	0.668
Placenta previa type			0.209
Pernicious	25	15	
Marginal	26	32	
Complete	62	64	
Gestational hypertension	9	3	0.072
Anemia	14	10	0.284
Twins	9	3	0.072
GDM	23	15	0.106
Eclampsia	2	2	1.00
Oligohydramnios	2	3	1.00
Premature delivery	64	46	0.002
IVF	9	11	1.00
Operative time	72.3 ± 45.7	61.0 ± 39.2	0.053

ICS, intraoperative cell salvage; GDM, gestational diabetes mellitus; IVF, *in vitro* fertilization.

Of the 102 ICS collections, 89 (87.3 %) were transfused and 13 (12.7 %) were wasted because of the lack of transfusion indications. Thirty-seven of the 89 patients receiving reinfusion of ICS blood also underwent ABT because of an insufficient volume of recovered blood. Of the 113 controls, 31 underwent ABT. The ABT rate was not significantly different between the ICS and control groups (36.3 vs. 27.4 %, p=0.19). The EBL was significantly higher in the ICS group compared with the control group [median (IQR): 550 (300, 800) mL vs. 400 (300, 600) mL, p=0.01] (Table 2). The overall ABT volume and the volumes of each blood component, including perioperative RBCs, frozen plasma, cryoprecipitate, and platelets, was also compared between the two groups, revealing no significant differences (p>0.05, Table 2). Intraoperative median (IQR) RBC value was significantly lower in the ICS group than in the control group (p=0.001; Table 2).

**Table 2:** Perioperative exposure to blood products of patients in the ICS and control groups.

	ICS (n=102)	Control (n=113)	p-Value
Estimated blood loss, mL	550 (300, 800)	400 (300, 600)	0.01
Overall ABT rate (n, %)	37 (36.3)	31 (27.4)	0.19
Overall ABT volume, mL	478.1 ± 1,207.2	449.5 ± 1,080.3	0.855
Red blood cell			
Perioperative, U	3.76 (3.6, 4.01)	3.75 (3.52, 3.95)	0.37
Intraoperative, U	3.73 (3.36, 4.02)	3.48 (3.15, 3.85)	0.001
Frozen plasma			
Perioperative, mL	400 (200, 800)	400 (400, 800)	0.67
Intraoperative, mL	400 (200, 800)	400 (400, 800)	0.42
Cryoprecipitate			
Perioperative, mL	4 (2.5, 8)	7 (4, 8)	0.16
Intraoperative, mL	5 (4, 9)	7 (4, 11)	0.39
Platelet			
Perioperative, mL	1 (1, 3)	1 (1, 1)	>0.99
Intraoperative, mL	1 (1, 1)	1 (1, 1)	>0.99

ICS, intraoperative cell salvage; ABT, allogeneic blood transfusion.

Linear regression analysis was performed to determine the factors associated with ABT volume. The results showed that EBL (p=0.000, 95 %CI=0.358–0.825) and operative time (p=0.003, 95 %CI=2.702–13.241) were independent factors (Table 3). Subsequently, patients in the ICS and control groups were stratified according to the mean EBL and operative time. As a result, the overall ABT rate, median (IQR) ABT volume and median (IQR) RBC values were found to be significantly different between the ICS group and the control group for patients with EBL <800 mL (p<0.05). Additionally, the median (IQR) RBC value was significantly lower in the ICS group than in the control group for patients with EBL >800 mL (Table 4).

**Table 3:** Results of linear regression analysis.

		Regression coefficient	Standard error	T	p-Value	95 % CI	
						Lower limit	Upper limit
ABT volume	Estimated blood loss	0.591	0.117	5.035	0.000	0.358	0.825
	Operative time	7.971	2.648	3.011	0.003	2.702	13.241
Postoperative Hb	Autologous transfusion volume	−0.018	0.009	−2.046	0.044	−0.035	0.000
Postoperative FIB	Autologous transfusion volume	0.003	0.002	2.024	0.050	0.000	0.006

ABT, allogeneic blood transfusion; CI, confidence interval; Hb, hemoglobin; FIB, fibrinogen.

**Table 4:** Stratification of risk factors for allogeneic transfusion.

		Overall ABT rate (n, %)	Overall ABT volume, mL	RBCs, U	Frozen plasma, mL	Cry, mL	Platelet, mL
Estimated blood loss							
<800	ICS (n=81)	31 (38.3)	0 (0, 0)	0 (0, 0)	0 (0, 0)	0 (0, 0)	0 (0, 0)
	Control (n=94)	12 (12.8)	0 (0, 0)	0 (0, 0)	0 (0, 0)	0 (0, 0)	0 (0, 0)
	P-value	<0.001	0.03	0.03	0.07	0.07	>0.99
>800	ICS (n=21)	18 (75)	406.00 (0, 713.00)	4.00 (0, 8.00)	400.00 (200.00, 1,200.00)	4.00 (2.00, 12.00)	0 (0, 0)
	Control (n=19)	19 (100)	408.00 (404.00, 816.00)	8.00 (4.00, 12.00)	800.00 (400.00, 800.00)	6.00 (0, 8.00)	0 (0, 0)
	P-value	0.233	0.31	0.01	0.32	0.96	0.98
Operative time							
< 67 min	ICS (n=61)	10 (38.3)	0 (0, 0)	0 (0, 0)	0 (0, 0)	0 (0, 0)	0 (0, 0)
	Control (n=89)	13 (12.8)	0 (0, 0)	0 (0, 0)	0 (0, 0)	0 (0, 0)	0 (0, 0)
	P-value	0.820	0.70	0.43	0.34	0.51	>0.99
>67 min	ICS (n=41)	27 (65.9)	2.00 (0, 408.00)	2.00 (0, 4.00)	200.00 (0, 600.00)	2.00 (0, 4.00)	0 (0, 0)
	Control (n=24)	18 (75)	355.50 (0, 662.50)	5.00 (1.00, 11.00)	400.00 (0, 800.00	2.00 (0, 8.00)	0 (0, 0)
	P-value	0.580	0.51	0.11	0.39	0.99	0.70

ABT, allogeneic blood transfusion; RBCs, red blood cells; ICS, intraoperative cell salvage; Cry, cryoprecipitate.

The ABT costs of the two groups were also compared overall and by stratification based on the EBL and operative time. The results showed that the median (IQR) ABT costs were significantly higher in the ICS group in patients with an intraoperative EBL <800 mL who underwent surgeries <67 min, which may be attributed to the higher cost of unused salvaged blood in some cases. However, no significant difference in ABT costs was observed in patients with intraoperative EBL >800 mL, or those who underwent surgeries >67 min (Table 5).

**Table 5:** Cost of treating patients in the ICS and control groups.

	Group	Cost, RMB	p-Value
Overall	ICS (n=102)	2,879.2 ± 2,748.6	<0.001
	Control (n=113)	867.0 ± 2,096.7	
Estimated blood loss			
<800	ICS (n=81)	1,836.00 (1,836.00, 1,836.00)	0.001
	Control (n=94)	0 (0, 0)	
>800	ICS (n=21)	3,968.00 (2,556.00, 5,758.00)	0.39
	Control (n=19)	4,015.00 (1,540.00, 5,510.00)	
Operative time			
< 67 min	ICS (n=61)	1,836.00 (1,836.00, 1,836.00)	0.001
	Control (n=89)	0 (0, 0)	
> 67 min	ICS (n=41)	3,053.00 (1,836.00, 4,561.00)	0.10
	Control (n=24)	2,120.00 (340.00, 4,875.00)	

ICS, intraoperative cell salvage.

Routine blood and coagulation function tests (D-dimer and fibrinogen) were performed before and 24 h after surgery in all patients. The results indicated that there was no significant difference in the median (IQR) of preoperative Hb, HCT, or D-dimer levels between the two groups; however, the median (IQR) postoperative Hb and HCT levels were significantly higher in the ICS group than in the control group (p<0.05) (Table 6), indicating that ABT may be better for maintaining oxygen supply and promoting rapid recovery from surgery. Regression analysis further revealed a negative correlation between ABT volume and postoperative level of Hb (r=−0.018, p=0.044) and a positive correlation between ABT volume and postoperative fibrinogen level (r=0.003, p=0.050) (Table 3).

**Table 6:** Perioperative indicators of patients in the ICS and control groups.

		ICS group (n=102)	Control group (n=113)	p1-Value
Routine blood	Preoperative Hb	112 (106, 118)	112 (106, 118)	0.67
	Postoperative Hb	108.5 (100, 118)	102 (94, 111)	0.004
	P2	<0.001	<0.001	
	Preoperative HCT	34.2 (32.5, 35.7)	33.6 (32.1, 35.5)	0.64
	Postoperative HCT	33.4 (30.4, 35.8)	31.5 (28.1, 34.4)	0.004
	P2	<0.001	<0.001	
	Preoperative RBCs	4 (2, 5)	4 (4, 10)	0.046
	Postoperative RBCs	2 (2, 4)	4 (4, 8)	0.004
	P2	0.006	0.02	
Coagulation	Preoperative D-D	2.00 (1.49, 2.78)	2.11 (1.23, 2.59)	0.65
	Postoperative D-D	4.65 (2.71, 8.99)	4.17 (1.97, 8.68)	0.32
	P2	<0.001	<0.001	
	Preoperative FIB	4.2 (3.7, 4.7)	3.9 (3.5, 4.3)	0.011
	Postoperative FIB	4.3 (3.7, 4.8)	3.7 (2.8, 4.5)	0.004
	P2	0.035	0.39	
Newborns	One-min apgar score	9 (9, 9)	9 (9, 9)	0.054
	5-min apgar score	9 (9, 9)	9 (9, 9)	0.017

ICS, intraoperative cell salvage; Hb, hemoglobin; HCT, hematocrit; RBCs, red blood cell; D-D, D-dimer; FIB, fibrinogen; P1, p-value between ICS, and control groups; P2, p-value between preoperative and postoperative comparisons.

## Discussion

ICS and retransfusion are commonly used methods for perioperative blood management. Theoretically, the transfusion of RBCs obtained from the cell salvage system may eliminate the need for additional autologous or ABT [18]. However, several studies failed to show a clear efficacy and cost-effectiveness [20], including in patients undergoing cesarean section [16], 17]. The present study further confirmed the value of ICS for patients with PP during cesarean section, which has only rarely been independently investigated [18]. In this cohort, ICS did not significantly reduce the overall ABT rate compared with the control group (36.3 vs. 27.4 %, p=0.19), despite the ICS group exhibiting a significantly higher median EBL (550 vs. 400 mL, p=0.01). Stratified analyses further revealed that ICS conferred distinct benefits in subgroups stratified by EBL and operative time, and patients in the ICS group achieved significantly higher postoperative Hb and HCT levels than controls (p<0.05).

In contrast to the studies of Zeng et al. [16] and Liu et al. [21], our results revealed that the use of ICS did not reduce the overall requirement for ABT. Nevertheless, a trend toward lower median allogeneic RBCs transfusion was observed in the ICS group among patients with operative time >67 min, although the difference was not statistically significant. This decrease in blood product utilization did not translate into significant cost saving for each patient, which was consistent with the study of Zeng et al. [20], but not Cresswell et al. [15]. These differences in findings may be attributed to the following reasons: 1) differences in the sample size; 2) a lack of clear guidelines for ABT in different countries, meaning that the use of ICS may be determined based on the experience of surgeons; 3) the expense standard may be different; and 4) the equipment used for ICS varied, with instruments including the Cell Saver Elite (Haemonetics) [18], Cell Saver 5+ (Haemonetics) [16], 20], Cell Saver 5 (Haemonetic) [14], and the cell saver Xtra (Sorin) [12] all being applied. Compared with the others, the cell saver Xtra may be more appealing for its performance in producing high HCT levels in the washed RBC volume, with a higher RBC recovery rate and fat elimination rate [22], 23]. In our study, we used the cell saver Xtra, which may be one reason for the higher postoperative levels of Hb and HCT in our patients with ICS re-transfusion compared with the study of Liao et al. [24]. The higher postoperative levels of Hb and HCT may have resulted in an increase in the oxygen-carrying capacity and tissue oxygen delivery that may have been beneficial to patient recovery and could have contributed to the shorter duration of postoperative hospital stay. This economic benefit may be another potential advantage for ICS [18].

This study has several limitations which should be noted. First, its retrospective design may have led to a selection bias. For example, the lower number ABT patients in the control group may have been due to the underestimation of EBL in 2014 and 2015 in our hospital. Further, patients in the ICS group may have been more complex, because some confounding factors may not have been completely eliminated. In addition, some outcomes may have been missing; therefore, a comparison could not be performed. Second, no protocol was applied to determine whether ICS should be administered and whether salvaged blood should be re-transfused. Third, this study had a single-center design, and the sample size was relatively small. Thus, further multicenter randomized controlled trials are necessary to determine the benefits of ICS in patients with PP during cesarean sections.

## Conclusions

Overall, our findings suggest that ICS may be recommended for patients with PP who undergo cesarean section for more than 1 h. Although ICS could not reduce ABT costs, its effectiveness in improving postoperative levels of Hb and HCT may be beneficial to ensure the rapid recovery of patients after surgery.
